# MosSCI and Gateway Compatible Plasmid Toolkit for Constitutive and Inducible Expression of Transgenes in the *C. elegans* Germline

**DOI:** 10.1371/journal.pone.0020082

**Published:** 2011-05-26

**Authors:** Eva Zeiser, Christian Frøkjær-Jensen, Erik Jorgensen, Julie Ahringer

**Affiliations:** 1 The Gurdon Institute and Department of Genetics, University of Cambridge, Cambridge, United Kingdom; 2 Department of Biomedical Sciences and Danish National Research Foundation Centre for Cardiac Arrhythmia, University of Copenhagen, Copenhagen, Denmark; 3 Howard Hughes Medical Institute, Department of Biology, University of Utah, Salt Lake City, Utah, United States of America; Virginia Tech, United States of America

## Abstract

Here we describe a toolkit for the production of fluorescently tagged proteins in the *C. elegans* germline and early embryo using Mos1-mediated single copy insertion (MosSCI) transformation. We have generated promoter and 3′UTR fusions to sequences of different fluorescent proteins yielding constructs for germline expression that are compatible with MosSCI MultiSite Gateway vectors. These vectors allow tagged transgene constructs to be inserted as single copies into known sites in the *C. elegans* genome using MosSCI. We also show that two *C. elegans* heat shock promoters (*Phsp-16.2* and *Phsp-16.41*) can be used to induce transgene expression in the germline when inserted via MosSCI transformation. This flexible set of new vectors, available to the research community in a plasmid repository, should facilitate research focused on the *C. elegans* germline and early embryo.

## Introduction

Transgene silencing in the *C. elegans* germline has hampered research in this tissue and the early embryo. Such silencing is caused by repetitive transgene arrays that form upon injection of DNA in the gonad. The creation of more “complex” extrachromosomal arrays through inclusion of fragmented genomic DNA, and the use of microparticle bombardment for low copy number insertions, finally allowed germline expression of transgenes [1], [2]. However, bombardment is labour intensive and complex extrachromosomal arrays are often still silenced. Furthermore, both methods frequently yield transformants with multiple transgene copies, which can have disadvantageous dosage related effects.

Recently, the Mos1 mediated Single Copy Insertion (MosSCI) method was developed to insert single copies of transgenes into defined sites in the genome of *C. elegans* [3]. Single copy insertion overcomes problems of variable gene dosage and silencing of extrachromosomal or integrated arrays in the germline. This technique is based on the MosTIC technique [4]. It makes use of *C. elegans* strains harbouring single *Drosophila* Mos1 transposon insertions at annotated sites in the genome. Following the heterologous expression of the Mos1 transposase, the transposon is excised from the genome, leaving a site-specific double strand break. If excision is carried out in the presence of a vector containing genomic DNA sequences that flank the Mos1 insertion site, template-directed repair can occur via homologous recombination, leading to integration of sequences cloned between the Mos1 flanking genomic DNA sequences. A library of strains containing Mos1 insertions was generated by the NEMAgenetag consortium, providing a large number of potential sites of integration [5]. Currently four Mos1 insertion strains with corresponding integration vectors have been validated for MosSCI and made available to the community [3], [6].

The advantageous features of single copy insertion motivated us to explore the use of MosSCI generated transgenes for studies in the germline and early embryo. We designed a vector toolkit of germline compatible constructs compatible with the MultiSite Gateway system. MultiSite Gateway technology enables users to fuse up to four different sequences captured in Gateway recombination frames, via a one step reaction into a single fusion sequence. The system guarantees that the fragments fuse in a defined orientation and order designated by the recombination frames. Prior to the recombination reaction each of the sequences of interest are subcloned into the appropriate MultiSite Gateway vector yielding entry clones; these are then combined into a destination vector yielding an expression clone. From a collection of entry clones, different combinations of fragments can be chosen which is pivotal for the flexibility represented by the MultiSite Gateway system. The system has been widely adopted in the *C. elegans* community and several genome scale resources such as the promoterome [7], ORFeome [8] and 3′UTRome [9] were generated that are compatible with MultiSite Gateway.

The plasmids of the toolkit are entry clones designed for the generation of expression clones using three sequences: a 5′, a middle and a 3′ fragment. The toolkit allows both N-terminal and C-terminal fluorescent protein tags; we provide promoter and promoter fusions as 5′ fragments for N-terminal tagging and 3′ UTR fusions as 3′ fragments for C-terminal tagging. The middle fragment contains the ORF of interest, provided by the user. The destination vector has sites for recombination of these three elements flanked by genomic sequences adjacent to a Mos1 site of interest; our reagents are compatible with all published MosSCI sites [3], [6]. Using an appropriate combination of 5′ and 3′ constructs with the ORF of one's choice and one of the available destination vectors, it is easy to generate a construct that will integrate at a target site in the genome and mediate constitutive expression of an N- or C-terminal fluorescently tagged recombinant protein in the germline or early embryo.

## Results and Discussion

### *mex-5* promoter and *tbb-2* 3′UTR constructs for constitutive expression in the germline

**Figure 1:**
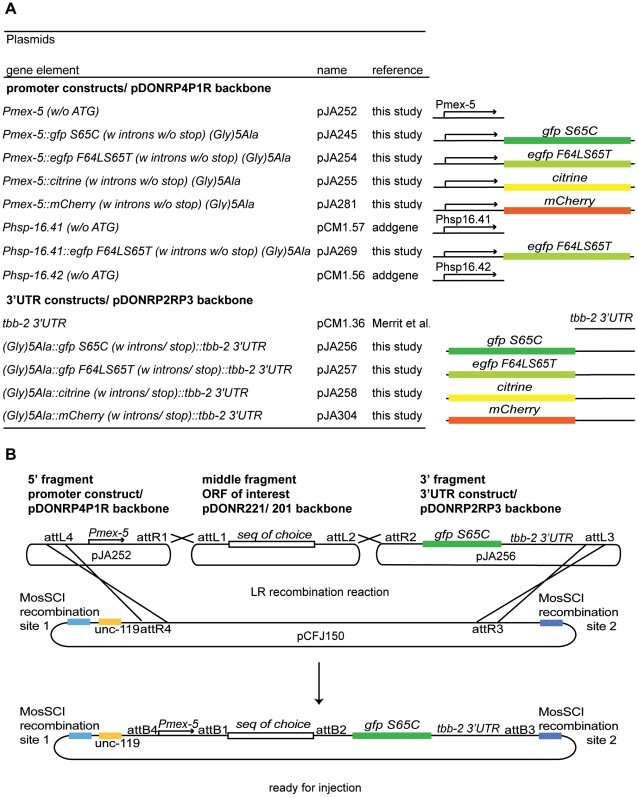
Plasmids for germline expression in *C. elegans*. (A) Descriptions and diagrammatic representations of promoter and 3′UTR constructs ready for use in MultiSite Gateway cloning. (B) Schematic diagram depicting the generation of an expression clone using MultiSite Gateway cloning mediated by the LR enzyme using 5′ and 3′ fragment plasmids listed in (A), a user's ORF for the middle fragment, and a MosSCI compatible destination vector. The ORF of choice needs an ATG for C-terminal tag fusions in combination with the *mex-5* promoter construct pJA252 and optimally should contain a stop codon for N-terminal tag fusions. The destination vector pCFJ150 contains genomic regions flanking the ttTi5605 Mos1 insertion to generate MosSCI inserts at this locus (carried in strain EG4322).

As regulatory 5′ element for constitutive transgene expression in the germline we chose the *mex-5* promoter. A small 486 bp *mex-5* promoter fragment had previously been shown to drive robust germline specific gene expression in strains made by microparticle bombardment [10]. We generated a set of 5′ entry clones containing the *mex-5* promoter fused to *gfp* (S65C), *egfp* (F64LS65T), *citrine* and *mCherry* (Figure 1). In addition, we also generated a 5′ entry clone containing the *mex-5* promoter lacking a start codon to allow use of the start ATG in an ORF clone.

We based our 3′ constructs on the *tbb-2* 3′UTR, which had been shown to be permissive for expression in all cell stages of the germline and in embryos [10]. We fused the *tbb-2* 3′UTR to sequences of *gfp* (S65C), *egfp* (F64LS65T), *citrine* and *mCherry*. An untagged *tbb-2* 3′UTR clone (pCM1.36) is already available [10].

Expression of transgenes in *C. elegans* is promoted by the presence of introns or syntrons (artificial introns) [11]. The sequences that code for fluorescent proteins in the fusion constructs of the toolkit all contain syntrons, which should be advantageous for production of recombinant protein if a cDNA middle entry clone is used to generate the transgene. We also designed our constructs such that the linker (Gly)_5_Ala separates the fluorescent protein from its fusion partner in order to avoid possible negative steric interactions. The linker is additionally elongated by the sequence of the *att* recombination site that is generated in the MultiSite Gateway reaction.

Users of the toolbox can place a fluorescent fusion protein at the N-terminus using a *mex-5* promoter/fluorescent protein gene fusion, the ORF of choice, and the *tbb-2* 3′UTR. C-terminal fusions are created using the *mex-5* promoter, the ORF of choice, and a fluorescent protein gene/*tbb-2* 3′UTR fusion. The *tbb-2* 3′UTR fusion constructs can also be combined with other (non-germline specific) promoters for expression of C-terminally tagged proteins in other tissues. Combining these sets with a MosSCI destination vector in a Gateway reaction generates a construct ready for injection into the appropriate Mos1 harbouring strain.

### Germline expression of transgenes

**Figure 2:**
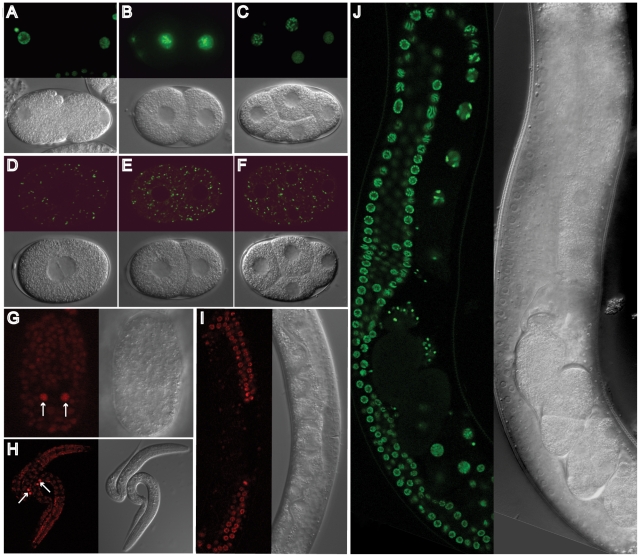
Expression of transgenes generated using toolbox plasmids. (A–C) *Pmex-5/his-58/egfp::tbb-2 3′UTR* expression produced signal marking chromatin in embryos of strain JA1522. (D–F) *Pmex-5/manS/citrine::tbb-2 3′UTR* expression produced signal marking the Golgi apparatus in embryos of strain JA1534. (G–I) *Pmex-5::mCherry/his-58/tbb-2 3′UTR* (strain JA1527) (G) late embryo and (H) L1 animals showing high signal in germline precursors Z2 and Z3 (arrows), lower signal in somatic nuclei (I) fluorescence signal in the germline of L4 stage. (J) *Pmex-5/his-58/egfp::tbb-2 3′UTR* JA1522 adult, HIS-58-EGFP can be detected in the gonad, oocytes, sperm and embryos. In general, signals were brighter at 25°C than at 15°C, and the signal produced by GFP S65C seems to have a better photostability than EGFP F64LS65T (not shown) [14].

In order to validate the 5′ and 3′ entry clones of the toolkit for germline expression, we generated and integrated a series of transgenes fusing GFP, EGFP, Citrine, or mCherry as N-terminal or C-terminal fusions (see methods); representative examples for the histone HIS-58 and a portion of the Golgi enzyme AMAN-2, are shown in Figure 2. All fusion proteins were visible in all regions of the hermaphrodite germline and in embryos (Figure 2 and data not shown). Fluorescence was high in early embryos and then declined in most cells during embryogenesis, presumably through degradation. In the hermaphrodite germline, fluorescence remained continuously high throughout development (Figure 2G, H, I). We also observed *mex-5* promoter driven transgene expression in the male germline (data not shown).

### Heat shock induced expression in the germline driven by P*hsp-16.2* and P*hsp-16.41*

The *mex-5* promoter allows constitutive expression of transgenes in the germline. However, inducible expression is needed when proteins might have a toxic effect. The heat shock promoters P*hsp-16.2* and P*hsp-16.41* have been used extensively for ectopic induction of gene expression in somatic cells, but such transgenes have failed to drive observable fluorescent fusion protein expression in the germline [12]. A recent report used *hsp-16.2* promoter fusions to generate germline phenotypes suggesting that this promoter is active in the germline, but did not characterize its activity [13].

**Figure 3:**
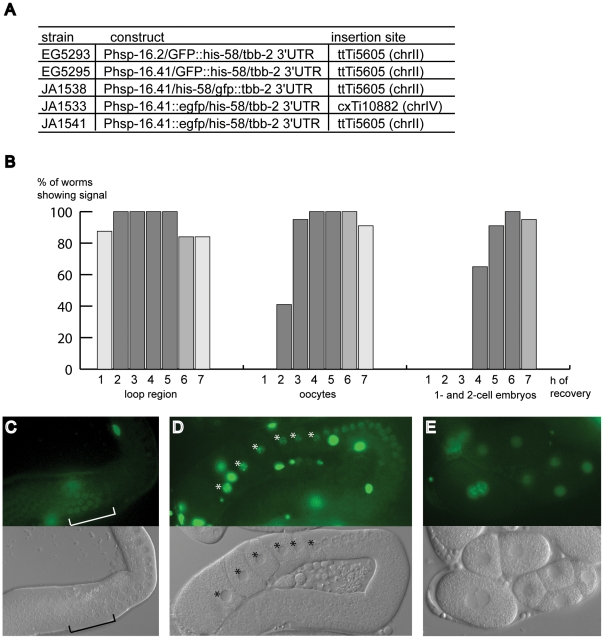
Activity of heat shock promoters in the *C. elegans* germline. (A) MosSCI strains generated for heat shock experiments. (B) Time course analyses of *Phsp-16.41/gfp;:his-58/tbb-2 3′UTR* (strain EG5295). Different shades of gray indicate rough quantification of average intensity levels of signals observed at indicated time points after heat shock. Darker shades indicate a stronger signal. Data were collected in two independent experiments observing 7–13 samples per stage at each time point; embryos were assessed starting from 3 h of recovery. Regions scored are shown in (C–E). (C) GFP-HIS-58 fluorescence observed close to the loop region of the gonad at 1 h after recovery from heat shock. (D) Fluorescence in oocyte nuclei (stars) at 4 hours post heat shock. (E) Fluorescence in embryonic nuclei at 4.5 h after heat shock.

To test the activity of heat shock promoters in the germline when present as single copy insertions, we generated constructs containing the *hsp-16.2* or *hsp-16.41* promoter and *tbb-2 3′UTR* regulating the expression of *gfp* tagged *his-58* and integrated them using MosSCI. Five strains were generated differing in promoter, tag sequences and its location and integration site (Figure 3A). All transgenes were expressed in soma, germline and embryos following heat shock. Somatic expression was much stronger than that in the germline and we observed variation in the intensity of expression in the germline. Additionally, the signal from constructs made with EGFP fused to Phsp-16.41 (strains JA1533 and JA1541) was weaker than the signal from GFP constructs. We do not know the cause of this difference but others have reported that GFP S65C performs better in *C. elegans* than EGFP F64LS65T [14].

We examined the timing of appearance of transgene expression using the *hsp-16.41* promoter strain EG5295. We subjected adult hermaphrodites to a one hour heat shock at 33°C followed by recovery at 20°C, and observed the animals and their progeny at one hour intervals. Immediately following the heat shock, onset of GFP fluorescence was visible only in the soma. After one hour of recovery, weak nuclear localised GFP signal could be seen in proximal germ cell nuclei near the loop region (Figure 3C). GFP signal was visible in oocytes after two hours, and then in embryos after four hours (Figure 3D and E). The intensity of the signal also grew stronger between one and three hours following recovery (Figure 3B). After six hours, signal in the gonad began to diminish (Figure 3B). Similar results were seen using the *hsp-16.2* promoter (data not shown).

In summary, we have generated a flexible set of constructs to produce fluorescent fusions to an experimenter's protein of interest in the *C. elegans* germline, using MultiSite Gateway technology and MosSCI transgenesis. The toolbox constructs, available through Addgene (http://www.addgene.org) should be a valuable resource for studying germline and early embryo development.

## Methods

### Plasmid construction

Entry clones were generated using the MultiSite Gateway Three-Fragment Vector Construction Kit (Invitrogen). Inserts were amplified from genomic DNA or plasmid templates using the High Fidelity Phusion Polymerase (Finnzymes, Espoo, Finland). PCR products were recombined into pDONRP4-P1R, pDONR221 or pDONRP2R-P3 using the BP clonase (Invitrogen). Inserts were verified by sequencing. To generate the expression clones a set of entry clones were fused into either pCFJ150 or pCFJ201 using the LR clonase II (Invitrogen). Resulting plasmids were verified by restriction digest. Toolkit plasmids (see Figure 1) are available from Addgene (http://www.addgene.org).

### Creation of toolkit plasmids

pDONRP4-P1R backbone (5′ entry clones): **pJA245**: *Pmex-5::gfp::(Gly)_5_Ala* (GFP 65C); **pJA254**: *Pmex-5::egfp::(Gly)_5_Ala* (EGFP 64L 65T); **pJA255**: *Pmex-5::citrine::(Gly)*_5_ *Ala* (Citrine 203Y 221K); **pJA269**: *Phsp-16.41::egfp::(Gly)_5_Ala* (EGFP 64L 65T); **pJA281**: *Pmex-5::mCherry::(Gly)*_5_ *Ala*

The promoter of *mex-5* was amplified from genomic DNA, and fluorescent protein ORFs (containing syntrons) were from the following: *gfp 65C* from pPD95.02 (Fire Lab Vector Kit, June 1995), *egfp 64L 65T* from pPD104.53 (Fire Lab 1997 Vector Supplement, February 1997), *citrine 203Y 221K* a kind gift from Stefan Eimer, (CMPB, ENI, Goettingen), *mCherry* a kind gift from Karen Oegema (Ludwig Institute for Cancer Research, La Jolla). Sequence encoding a (Gly)_5_Ala spacer was added 3′ to the fluorescent protein sequence. The promoter and fluorescent protein sequences were fused via PCR stitching, with the outside primers containing attB4 and attB1 sites to allow recombination into pDONRP4-P1R.

pDONRP2R-P3 backbone (3′ entry clones): **pJA256**: (Gly)_5_Ala::gfp::tbb-2 3′UTR (GFP 65C); **pJA257**: (Gly)_5_Ala::egfp::tbb-2 3′UTR (EGFP 64L 65T); **pJA258**: (Gly)_5_Ala::citrine::tbb-2 3′UTR (Citrine 203Y 221K); **pJA304**: (Gly)_5_Ala::mCherry::tbb-2 3′UTR

The *tbb-2 3′UTR* was amplified from pCM1.36 [10] and fluorescent protein ORFs amplified from the sources described above. Sequence encoding a (Gly)_5_Ala spacer was added 5′ to the fluorescent protein sequence. The fluorescent protein ORF and *tbb-2 3′UTR* sequences were fused by via PCR stitching, with the outside primers containing attB2 and attB3 sites to allow recombination into pDONRP2R-P3.

### Expression clones

**pJA274**: *Pmex-5/his-58/(Gly)_5_Ala::egfp::tbb-2 3′UTR*. An LR reaction was performed using pJA252, pJA257, pJA273 (containing the *his-58* ORF w/o stop codon) and pCFJ150. **pJA275**: *Pmex-5/manS/(Gly)_5_Ala::citrine::tbb-2 3′UTR*. An LR reaction was performed using pJA252, pJA258, pJA276 (containing the first 301 bp of *aman-2* genomic sequence (encoding the first 84aa) in pDONR221) [15], and pCFJ201. **pJA283**: *Pmex-5::mCherry::(Gly)_5_Ala/his-58/tbb-2 3′UTR*. An LR reaction was performed using pJA281, pCM1.36, pEM295 (containing the *his-58* ORF, a kind gift of Nic Lehrbach), and pCFJ201. **pJA286**: *Phsp-16.41::egfp::(Gly)_5_Ala/his-58/tbb-2 3′UTR*. An LR reaction was performed using pJA269, pEM295, pCM1.36 and pCFJ201. **pJA290**: *Phsp-16.41/his-58/(Gly)_5_Ala::gfp::tbb-2 3′UTR*. An LR reaction was performed using pCM1.57, pJA273, pJA256 and pCFJ150. **pJA296**: *Phsp-16.41::egfp::(Gly)_5_Ala/his-58/tbb-2 3′UTR*. An LR reaction was performed using pJA269, pEM295, pCM1.36 and pCFJ150. **pCFJ179**: *Phsp-16.2/gfp::his-58/tbb-2 3′UTR*. An LR reaction was performed using pCM1.56, pCM1.35, pCM1.36 and pCFJ150. **pCFJ180**: *Phsp-16.41/gfp::his-58/tbb-2 3′UTR*. An LR reaction was performed using pCM1.57, pCM1.35, pCM1.36 and pCFJ150.

### Strains made or used in this study

See Table 1.

**Table 1:** Strains made or used in this study.

Strain	Genotype	Expression clone
EG4322	*ttTi5605 II; unc-119(ed3) III*	none
EG5003	*cxTi10882 IV; unc-119(ed3) III*	none
EG5293	*oxIs446 [Phsp-16.2/gfp::his-58/tbb-2 3′UTR; cb-unc-119 (+)] II*	pCFJ179
EG5295	*oxIs448 [Phsp-16.41/gfp::his-58/tbb-2 3′UTR; cb-unc-119 (+)] II*	pCFJ180
JA1522	*weSi6 [Pmex-5/his-58/(Gly)_5_Ala::egfp::tbb-2 3′UTR; cb-unc-119(+)] II*	pJA274
JA1527	*weSi14 [Pmex-5::mCherry::(Gly)_5_Ala/his-58/tbb-2 3′UTR; cb-unc-119(+)] IV*	pJA283
JA1533	*weSi19 [Phsp-16.41::egfp::(Gly)_5_Ala/his-58/tbb-2 3′UTR; cb-unc-119 (+)] IV*	pJA286
JA1534	*weSi13 [Pmex-5/manS/(Gly)_5_Ala::citrine::tbb-2 3′UTR; cb-unc-119(+)] IV*	pJA275
JA1538	*weSi23 [Phsp-16.41/his-58/(Gly)_5_Ala::gfp::tbb-2 3′UTR; cb-unc-119 (+)] II*	pJA290
JA1541	*weSi26 [Phsp-16.41::egfp::(Gly)_5_Ala/his-58/tbb-2 3′UTR; cb-unc-119 (+)] II*	pJA296

### MosSCI transformation

MosSCI transformation was performed based on the protocol described in [3] (http://sites.google.com/site/jorgensenmossci/). The Mos1 insertion strains EG4322 or EG5003 were used for injection. Injection mixes contained pJL43.1 (50 ng/µl), pCJF90 (2.5 ng/µl), pCFJ104 (5 ng/µl), and the respective expression clone (50 ng/µl) in 20 mM potassium phosphate and 3 mM potassium citrate (pH 7.5). We note that although we were able to obtain transgenic strains expressing each of the constructs described, some apparent integration events did not result in detectable expression; we do not know the reason for this variability.

### Heat shock induced germline expression

Worms were grown at 15°C to young adult stage and then heat shocked incubating sealed plates for 1 h in a water bath at 33°C. Subsequently the plates were incubated at 20°C and groups of worms were observed at 1 h intervals for fluorescence signals in the germline and embryonic progeny. After heat shock, the GFP signal strength in the germline was significantly lower than in somatic cells. Therefore, to observe germline and embryo GFP signals, worms were cut open to release the gonad and embryos. Observations were made using the 63× oil objective on a Zeiss Axioplan 2 fluorescence microscope. For the time course assessment data were collected in two independent experiments observing seven to thirteen samples of the different stages per time point with a total number ranging between 16 and 23. Observation started at 1 h of recovery for the loop region and oocytes and at 3 h of recovery for embryos. The weak germline signals were classified qualitatively into two categories: + (just detectable) and ++ (easily detectable). This qualification was translated into three shades of gray for the chart in Figure 3. The lightest shade of gray was assigned to time points when fewer than a third of observed signals were ++, and the darkest shade of gray when more than two thirds were ++ signals. Time points when ++ signals made up more than one third but less than two thirds of signals were coloured with the intermediate shade of grey.
